# A combined encoder–transformer–decoder network for volumetric segmentation of adrenal tumors

**DOI:** 10.1186/s12938-023-01160-5

**Published:** 2023-11-08

**Authors:** Liping Wang, Mingtao Ye, Yanjie Lu, Qicang Qiu, Zhongfeng Niu, Hengfeng Shi, Jian Wang

**Affiliations:** 1 College of Computer Science and Technology, Zhejiang University of Technology, Hangzhou, Zhejiang China; 2 Zhejiang Lab, No. 1818, Western Road of Wenyi, Hangzhou, Zhejiang China; 3 Department of Radiology, Sir Run Run Shaw Hospital, Zhejiang University School of Medicine, Hangzhou, Zhejiang China; 4 Department of Radiology, Anqing Municipal Hospital, Anqing, Anhui China; 5 Department of Radiology, Tongde Hospital of Zhejiang Province, No.234, Gucui Road, Hangzhou, Zhejiang China

**Keywords:** Adrenal tumor volumetric segmentation, Adrenal metastases, Transformer, Computer-aided diagnostic tool

## Abstract

**Background:**

The morphology of the adrenal tumor and the clinical statistics of the adrenal tumor area are two crucial diagnostic and differential diagnostic features, indicating precise tumor segmentation is essential. Therefore, we build a CT image segmentation method based on an encoder–decoder structure combined with a Transformer for volumetric segmentation of adrenal tumors.

**Methods:**

This study included a total of 182 patients with adrenal metastases, and an adrenal tumor volumetric segmentation method combining encoder–decoder structure and Transformer was constructed. The Dice Score coefficient (DSC), Hausdorff distance, Intersection over union (IOU), Average surface distance (ASD) and Mean average error (MAE) were calculated to evaluate the performance of the segmentation method.

**Results:**

Analyses were made among our proposed method and other CNN-based and transformer-based methods. The results showed excellent segmentation performance, with a mean DSC of 0.858, a mean Hausdorff distance of 10.996, a mean IOU of 0.814, a mean MAE of 0.0005, and a mean ASD of 0.509. The boxplot of all test samples' segmentation performance implies that the proposed method has the lowest skewness and the highest average prediction performance.

**Conclusions:**

Our proposed method can directly generate 3D lesion maps and showed excellent segmentation performance. The comparison of segmentation metrics and visualization results showed that our proposed method performed very well in the segmentation.

## Background

The adrenal glands are important secretory organs that are positioned above the kidneys on both sides of the body. The adrenal glands can be divided into cortical and medullary layers, which mainly secrete adrenal corticosteroids, epinephrine, and norepinephrine, and play an important role in maintaining the normal functioning of body organs [1, 2]. Adrenal tumors are formed by abnormal proliferation of local adrenal tissue cells and are pathologically classified into benign tumors (expansive growth) and malignant tumors (invasive growth) according to their biological behavior [3–5]. Both benign and malignant tumor types can affect hormone secretion and lead to hypertension, hyperglycemia, and cardiovascular diseases [6–8].

Computed tomography (CT) is an effective tool for diagnosing adrenal tumors, because it is non-invasive, produces clear images, and has high diagnostic efficiency. Because the morphology of the tumor (mainly refers to the smoothness of tumor edge) and the clinical statistics (mainly refers to the difference in density and gray level within the tumor) of the tumor area are two crucial diagnostic and differential diagnostic features, precise segmentation of the tumor is essential. There have been many studies evaluating traditional methods for adrenal tumor segmentation, such as classifying CT images pixel-by-pixel with a random forest classifier [9], and applying a localized region-based level set method (LRLSM) to segment the region of interest (ROI) [10]. Some studies have proposed segmentation frameworks that combine various algorithms to obtain better segmentation performance [11–13]. These include the K-means singular value factorization (KSVD) algorithm, region growing (RG), K-means clustering, and image erosion.

In recent years, with the continuous improvement in computing power and the development of deep learning techniques [14–16], deep learning has been increasingly applied in the medical field. In medical image diagnosis, many techniques have been used to assist in the diagnosis of disease through the segmenting of organs of interest. For example, research studies include the segmentation of brain tumors [17], segmentation of liver and liver tumors [18], and segmentation of kidneys [19]. However, all of the above studies segmented relatively large organs or tumors, and there is still room for improvement in the segmentation performance for small organs and small-volume tumors. Some researchers proposed the use of a knowledge-assisted convolutional neural network (KaCNN) for the difficult task of small organ segmentation, with this combining traditional and deep learning methods. The proposed framework has two phases: in the first phase, localization to the ROI region is performed, then in the second phase, segmentation of the ROI is performed by the proposed KaCNN, with the segmentation then being transformed back into the initial space [20]. However, only some studies applied deep learning to adrenal tumor segmentation. Such as Bi and Parehe have proposed using convolutional neural networks (CNNs) to segment adrenal tumors [21, 22].

In this study, a medical image segmentation method based on an encoder–decoder structure combined with a Transformer is proposed to perform volumetric segmentation for CT enhanced images of adrenal metastases. Considering that the proportion of tumor area is very small compared with the whole CT image, on average, the tumor volume only accounts for 0.267% of the whole sample (calculated by including samples in this study), which is very large from the perspective of segmentation difficulty. Therefore, we choose the DSC as the loss function to cope with the unbalanced situation of the number of foreground and background voxels.

## Results

### Patient characteristics

A total of 182 patients in the age group of 29–80 years (SD: 10.67) with a diagnosis of adrenal metastases were included in the study. Of these, 133 were single tumors, and 49 were multiple tumors.

### Quantitative analysis of segmentation performance

We compared our proposed method with other methods that have achieved good performance in medical image segmentation tasks, such as 3D U-Net [19], TransBTS [17], ResUNet [23], UNet++ [24], Attention U-Net [25] and Channel U-Net [26]. As shown in Table 1, our proposed method achieved the best performance in the segmentation of adrenal metastases, with a DSC of 0.858, Hausdorff distance of 10.996, and IOU of 0.814. However, the other methods also showed excellent performance, and the performance measures for the proposed method were not significantly different to those of the other methods ($P>0.3, P>0.5, P>0.5, P>0.8, P>0.2, P>0.1$). Although the differences were not significantly different, this analysis does at least show that our proposed method is equally effective as the other methods evaluated in the tumor segmentation task.

**Fig. 1:**
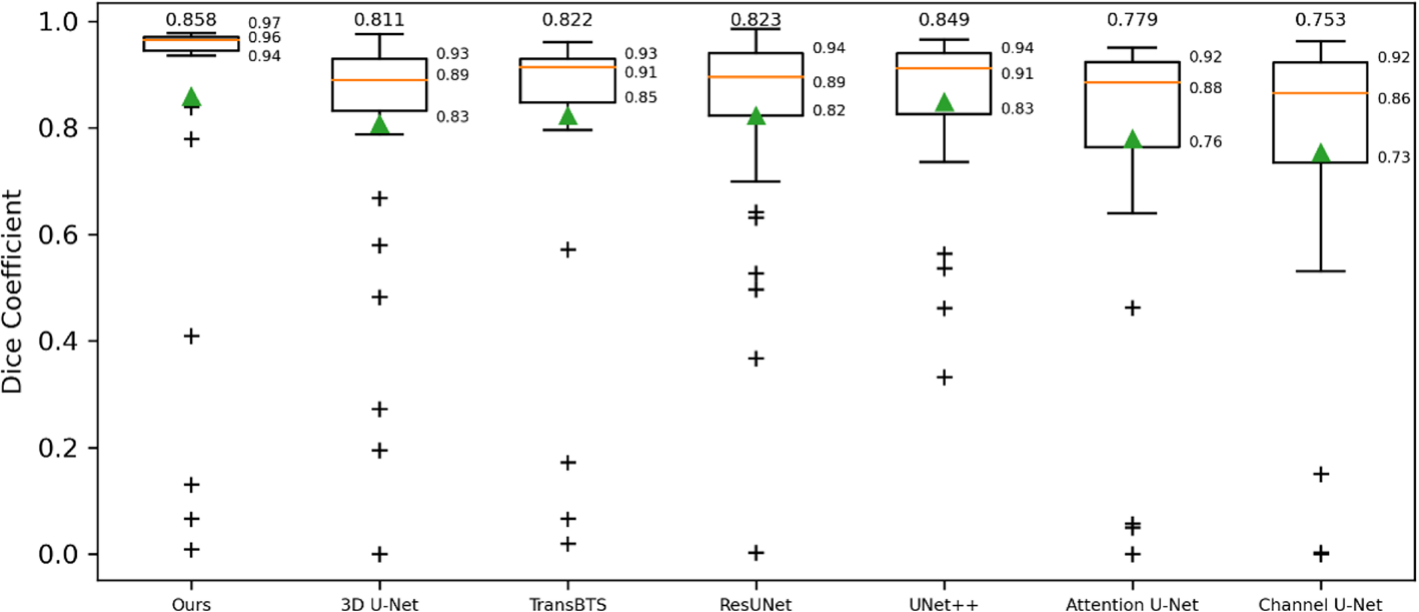
Comparison of the segmentation performance of different models for all test samples. The numbers at the top represent the mean values of each method (so is the green triangle). The upper, lower quartile, and median values are indicated on the right side of each box plot

Figure 1 shows the prediction performance statistics of the above seven methods on the test set. It can be observed that the best performance metric values were obtained with the proposed method. The prediction results of all methods showed different degrees of skewness. Moreover, there were more "outliers" with the proposed method than with the other methods, which means that although the overall performance of our proposed method was good, it did not perform well for the segmentation of complex samples with small lesions or artifacts.

### Visual comparison of segmentation results

**Fig. 2.**
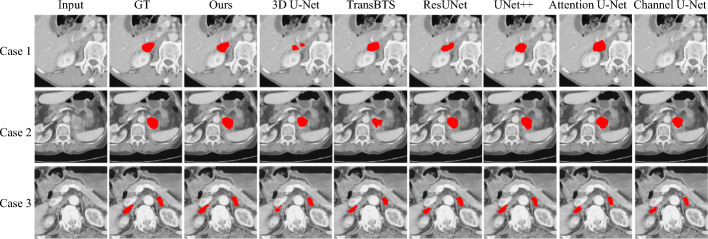
2D visualization of adrenal tumors. To see more details of segmentation results, we selected one slice from every case (note that the DSC of Channel U-Net is 0 in case 1)

2D visualization of some typical segmentation results is shown in Fig. 2. It can be seen that the method proposed in this paper is closest to the ground truth in the segmentation of lesions, both in terms of their borderline and shape. Due to the padding strategy, precise corner segmentations of every case have failed. It is discussed in [27] that padding may cause artifacts at the feature maps' borderlines, and these artifacts may cause the network to become confused.

Furthermore, we selected unilateral and bilateral adrenal tumors for 3D visualization and comparison in the test samples.

**Fig. 3:**
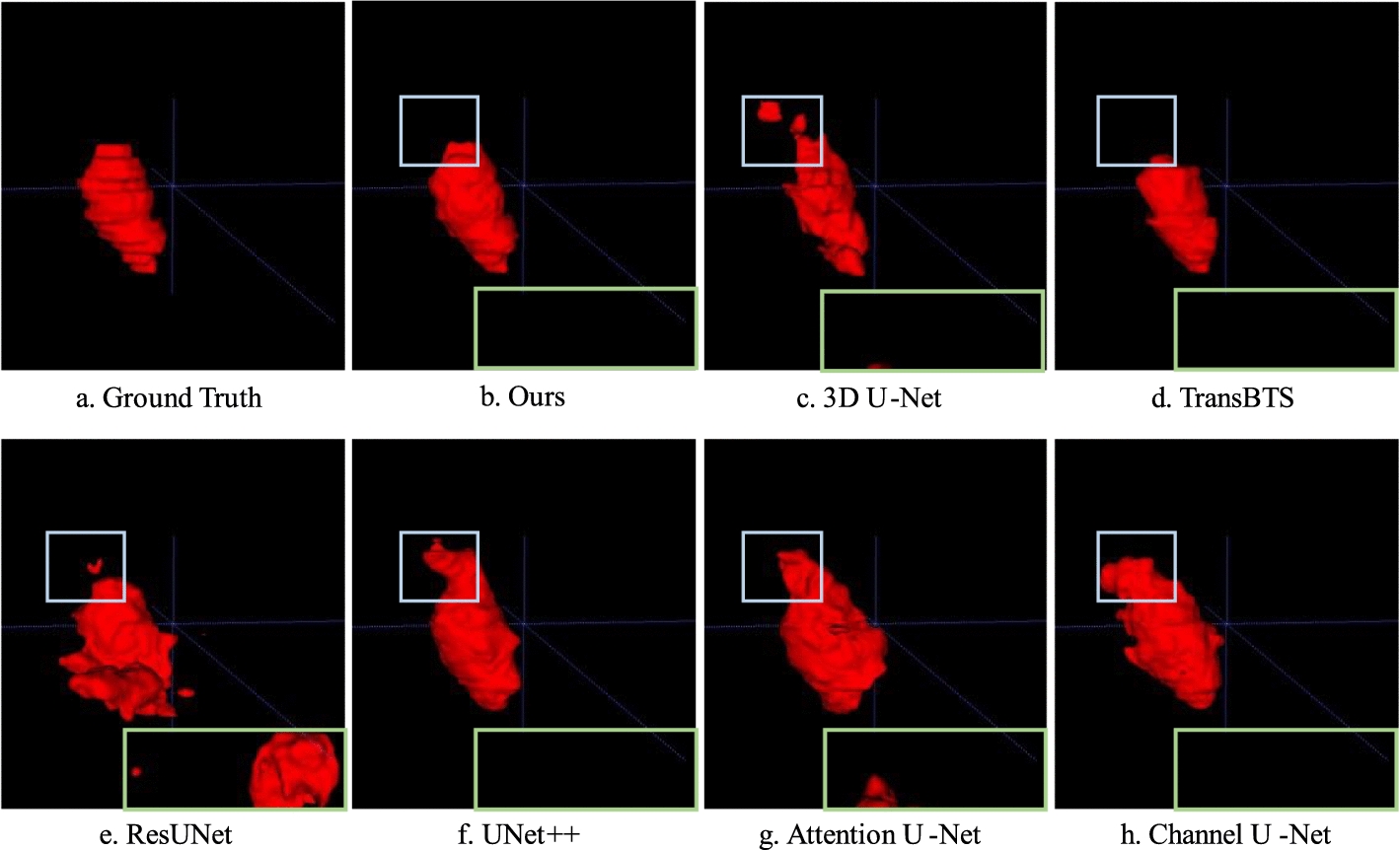
Comparison of the results of unilateral tumor segmentation. **a** Ground truth. **b** Our proposed method. **c** 3D U-Net. **d** The mask predicted by TransBTS is also close to the ground truth. **e** ResUNet mistakenly identified the organ on the other side as an adrenal tumor. **f** UNet++. **g** Attention U-Net also incorrectly identified the normal organ on the other side as a tumor. **h** Channel U-Net

The visualization results for the unilateral adrenal tumor are shown in Fig. 3, the sample size is $256 \times 256 \times 60$. It can be observed that our proposed method was morphologically closest to the ground truth mask, with a DSC of 0.951. The DSCs for 3D U-Net, TransBTS, ResUNet, UNet++, Attention U-Net, and Channel U-Net were 0.824, 0.856, 0.642, 0.797, 0.674, and 0.728, respectively.

**Fig. 4:**
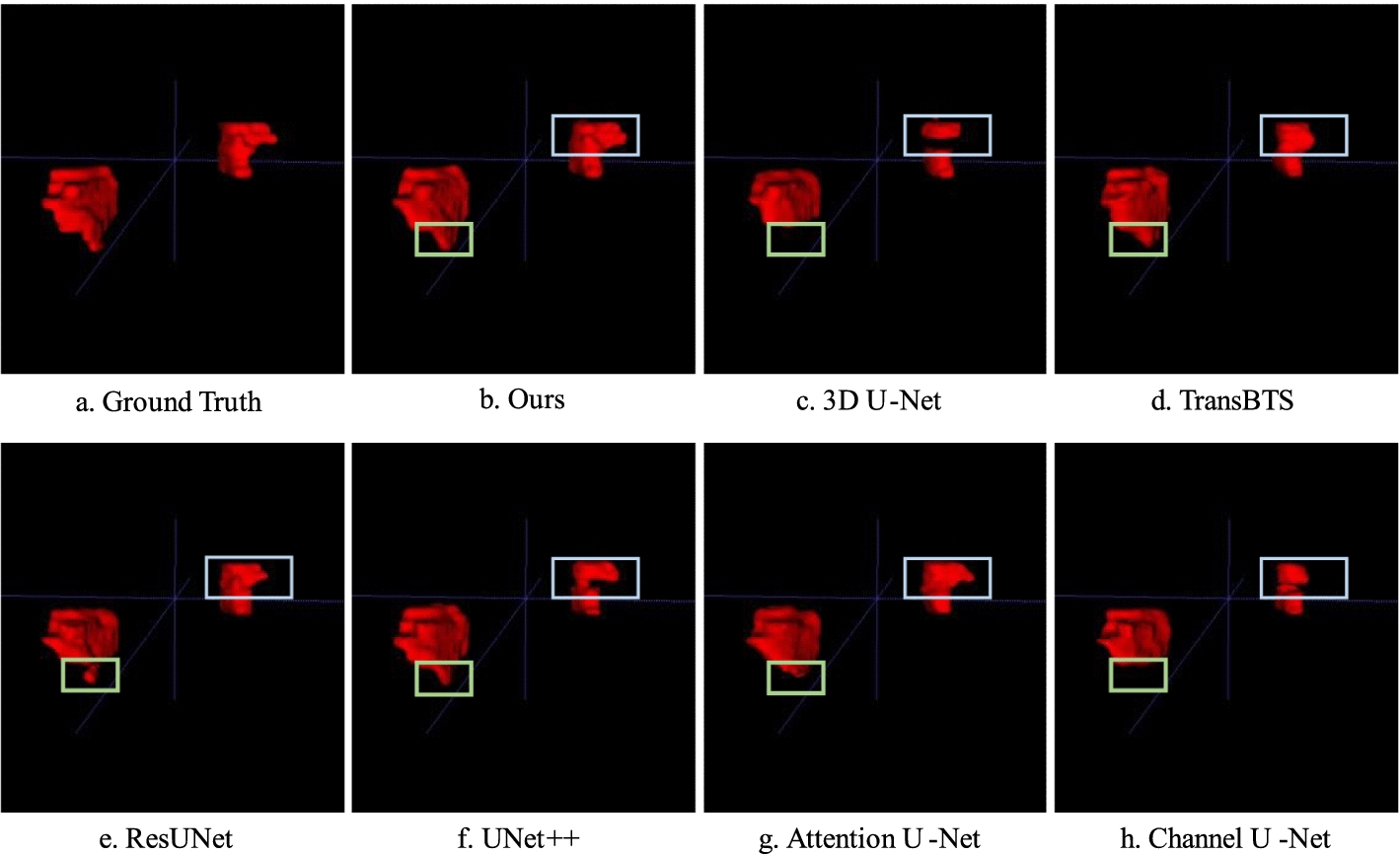
Comparison of bilateral tumor segmentation results. **a** Ground truth. **b** Our proposed method. **c** 3D U-Net mistakenly divided the right adrenal tumor into two parts. **d** TransBTS. **e** ResUNet. **f** UNet++. **g** Attention U-Net. **h** Channel U-Net

Figure 4 visually compares the test sample containing bilateral adrenal tumors. The sample size is $256 \times 256 \times 95$. The DSC of our proposed method was 0.947, whereas those of 3D U-Net, TransBTS, ResUNet, UNet++, Attention U-Net, and Channel U-Net were 0.857, 0.787, 0.868, 0.886, 0.866, and 0.766, respectively. Our prediction was closest to the ground truth label.

**Table 1:** Mean DSC, mean Haustoff distance, mean IOU, mean MAE, and mean ASD for the different models

Methods	DSC↑	Hausdorff distance↓	IOU↑	MAE↓	ASD↓
3D U-Net	0.811	29.957	0.721	0.0008	2.588
TransBTS	0.822	18.529	0.745	0.0007	1.927
ResUNet	0.823	21.908	0.736	0.0009	3.760
UNet++	0.849	13.297	0.761	0.0006	0.853
Attention U-Net	0.779	14.146	0.689	0.0010	2.341
Channel U-Net	0.753	15.724	0.660	0.0009	1.921
Ours	**0.858**	**10.996**	**0.814**	**0.0005**	**0.509**

### Ablation study

A series of ablation experiments were conducted to verify the effectiveness of each component of the proposed method. The major components are as follows: (a) ED/DSConv: the encoder–decoder/depthwise separable convolution network is the basic architecture of the entire network. We use the DSConv network to maintain a reasonable number of parameters when deepening the network, and this also ensures that the parameters will not be too large when later add the Transformer; (b) SC: the skip-connection structure is used for concatenate the feature maps generated by corresponding encoders and decoders; (c) TF: the transformer network has excellent modeling capabilities for establishing global dependencies. Table 2 shows the comparison results. It can be seen that DSC improves from 0.777 to 0.858, Hausdorff distance drops from 18.473 to 10.996, IOU improves from 0.690 to 0.814, MAE slightly decreases from 0.0006 to 0.0005, ASD drops from 1.544 to 0.509. All the evaluation metrics have different degrees of decline, proving our proposed method's effectiveness.

**Fig. 5:**
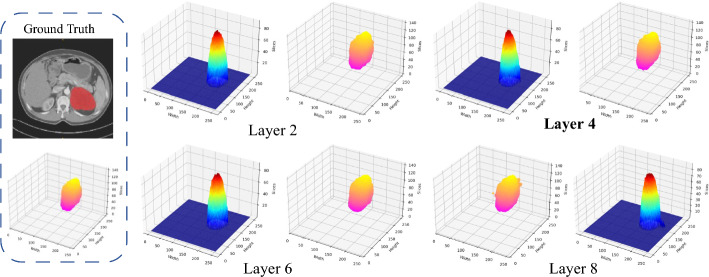
Last layer’s outputs of the model with different transformer layers. Except for Ground Truth, the left side of each subplot is the heat map, the color closer to red, the more model tends to judge this area as an abnormal tissue, and the right side is the tumor visualized in 3D (the color difference is to draw the 3D image in two-dimensional level)

We verified the influence of different transformer layers on the segmentation performance. Table 3 shows the experimental results of the Transformer layer of 2 layers, 4 layers, 6 layers, and 8 layers. The evaluation metrics of the model of 8 layers are even worse than that of 2 layers. To balance the number of model parameters and segmentation performance, we chose to use 4 Transformer layers for our proposed model. Figure 5 shows the heat map of the last layer's output of the model with different transformer layers. It can be observed that each model can correctly locate the tumor, but the model with 8 Transformer layers is not as good as the other three models at the borderline segmentation. Experiments show that the results will not be better when added more Transformer layers.

**Table 2:** Quantitative analysis of major components

ED/DSConv	SC	TF	DSC↑	Hausdorff distance↓	IOU↑	MAE↓	ASD↓
✓			0.777	18.473	0.690	0.0006	1.544
✓	✓		0.827	13.989	0.764	0.0005	1.738
✓	✓	✓	0.858	10.996	0.814	0.0005	0.509

## Discussion

The method proposed in this study extracts not only local features using a CNN, but also establishes global dependencies using a Transformer and uses deep separable convolution (DSConv) to reduce the number of parameters. Experiments demonstrated that our proposed method can accurately segment adrenal tumors, and that it outperformed mainstream medical image segmentation algorithms.

Traditional adrenal tumor segmentation methods have mostly used machine learning methods, such as random forest algorithms, regional level set algorithms, and K-means clustering algorithms. Although the above methods performed well in the segmentation of adrenal tumors, they were still subject to some limitations. For one thing, most studies collected only small quantities of data. In addition, the above methods are based on a two-dimensional image, whereas each CT scan actually includes three-dimensional image data, which may result in a time-consuming segmentation process. However, our proposed method can segment the whole lesion simultaneously based on the whole three-dimensional CT image set.

In recent years, image segmentation algorithms based on deep learning have achieved excellent performance in medical image processing. Segmentation of lung nodules [28–30] is a relatively mature application, and segmentation of brain tumors [31, 32], kidney [33], kidney tumors [34, 35], liver [36, 37], and liver tumors [38] are also research hotspots. There is a relevant study [39] that the authors employed a two-stage network to segment adrenal glands and evaluated the model with fivefold cross-validation, achieving excellent performance with a DSC of 0.874. However, the position and morphology of the adrenal gland are relatively fixed. The segmentation of adrenal tumors is more complex than that of adrenal glands because of the various locations and sizes and blurred contours of adrenal tumors. Bi combined CNN and RW to segment adrenal tumors and achieved a segmentation performance 0.729 (F1-Score) [21]. Parehe introduced a three-stage adrenal tumor segmentation and diagnosis network [22]. Although only a DSC of 0.690 was obtained, it did not affect the final diagnosis results. It is worth mentioning that this is the first time anyone has ever applied a Transformer to adrenal tumor segmentation. Because Transformer has achieved excellent results in natural language processing (NLP) tasks, it has regularly been applied to medical image segmentation. Transformer has excellent modeling capabilities and retains more spatial information and global features than convolutional neural networks. The method proposed in this paper takes advantage of both a convolutional neural network and a Transformer and achieved an average DSC of 0.858 in adrenal tumor segmentation.

For samples with bilateral adrenal tumors, early methods divided the bilateral tumors into two categories [9] or two stages to separate the bilateral tumors [13], indicating that the methods proposed in these studies needed additional location information to be provided manually. However, our proposed method will automatically learn relevant information about the image (such as shape and position information) in the feature extraction stage. The added position coding module can also ensure that the position information will be recovered after converting the image into a sequence. For image data preprocessing, most other methods have a set of complicated preprocessing processes that are very time-consuming to perform. With the powerful feature extraction capability of a convolutional neural network and a Transformer, the proposed method only requires superficial window level and window width adjustment and normalization processing.

To verify the superiority of our proposed method, we compared its performance with other mainstream medical image segmentation methods. The experimental results showed that our proposed method achieved the best performance, with a mean of DSC of 0.858, Hausdorff distance of 10.996, IOU of 0.814, MAE of 0.0005, and ASD of 0.509 in the segmentation of adrenal tumors. We selected three typical cases for two-dimensional visualization analysis and further selected unilateral and bilateral tumor test samples for three-dimensional visualization analysis. It can be intuitively observed that the segmentation result of our proposed method is closest to the ground truth both in shape and borderline. To verify the effectiveness of each component of the proposed method, we conducted extensive ablation studies, and the experimental results proved that each fundamental component contributed to the segmentation of tumors. We also further verified the influence of different transformer layers on the segmentation performance. The experimental results show that the optimal number of Transformer layers for the adrenal tumor segmentation task is 4, and the results will not be better when added more Transformer layers.

However, although our proposed method performed well, it also has some limitations. For example, the segmentation effectiveness of our proposed method was only verified on adrenal metastases, and the segmentation could have been better for very small tumors and those with very indistinct boundaries, which is an urgent problem to be solved in future work. Furthermore, the proposed method has not been tested on an external data set. Therefore, the performance generalizability has not been evaluated and should require further analysis.

## Conclusion

Our proposed segmentation method based on an encoder–decoder structure combined with a Transformer can effectively segment adrenal tumors, solving the problems of segmentation difficulties caused by the irregular location of adrenal tumors, generally small tumor volume, varying internal intensity, and blurred contours.

## Methods

### Patients selection

This retrospective study was approved by the ethics committee of Tongde Hospital of Zhejiang Province (approval number 2022-183). The data were anonymized, and the requirement for informed consent was waived.

A total of 182 patients diagnosed with adrenal metastases between January 2014 and August 2019 were considered for inclusion in this study. An experienced radiologist and a novice radiologist in the radiology department of our institution labeled the adrenal tumors on the CT images. Patients first received 100–120 ml of contrast material (Ultravist, Bayer Schering Pharma, Berlin, Germany) through an intravenous cannula inserted into a forearm vein, then underwent the multidetector CT examination. The original images were reconstructed from a 5-mm slice thickness into a 1.5-mm slice thickness. The parameters for the CT acquisitions were: 120 kV, 250–300 mA, 1.5–2.5 mm detector collimation, 1:1 table pitch, and 5-mm slice thickness. One author (J.W., abdominal imaging fellow) reviewed each sample manually to ensure that the diameter of each tumor was at least 10 mm in the long axis for the following two reasons: a. when the tumor is tiny, the boundary with normal adrenal tissue may not be apparent; b. If the tumor is tiny, it is more difficult to manually delineate the tumor, which will lead to lower consistency. In summary, we abandoned including tumor samples smaller than 10 mm.

### Data pre-processing

For each CT sample, the window level and width were adjusted to 40 and 300, respectively, to remove the information that was not important or irrelevant to tumor segmentation. Then, take one of the samples as an example; the sample size in the *x*, *y*, and *z* axes is $512 \times 512 \times 101$, respectively. The *x* and *y* axes were down-sampled to 0.5 times the original, and the *z*-axis was sampled to 1 mm slice thickness according to the spacing. The cubic interpolation method was used as the sampling method. Finally, a normalization operation is performed to simplify the computation and unify the dimensions.

### Proposed method

**Fig. 6:**
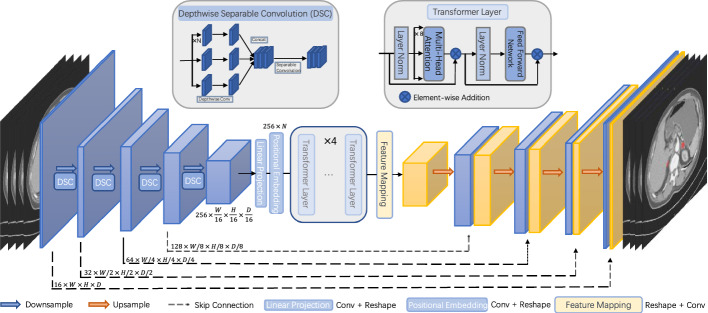
Overall architecture of the network

Our overall network architecture is shown in Fig. 6, with the encoder, decoder, and Transformer as the main framework. The encoder first down-samples the 3D input data while gradually extracting local features to generate a high-dimensional feature map. Then, the Transformer processes the high-dimensional feature map and establishes the global dependency. Finally, the new feature maps are connected with the previous feature maps which are down-sampled by the encoder at each step through the skip connection, and the segmentation results are obtained after up-sampling.

### Network encoder

For the volumetric medical image segmentation performed in this study, our input data were three-dimensional: $\mathrm{X}\in {\mathrm{R}}^{\mathrm{C}\times \mathrm{W}\times \mathrm{H}\times \mathrm{D}}$, where C is the channel, *W* is the width of each image item, *H* is the height of each image item, and *D* is the number of slices of the current input sample. In particular, the input image’s dimension of this model is $256\times 256\times 32$. The encoder stage performs down-sampling four times. For each down-sampling, the comprehensive data are down-sampled by a factor of two using the 3D CNN, and the channel dimension is changed to twice the original. The feature maps generated at each stage are temporarily saved. Because the receptive field of the shallow network is relatively small, the extracted features generally contain edge, texture, and angle information.

In contrast, because of its greater receptive field, the deeper network can extract more abstract information, i.e., deep semantic information. To extract deeper features while ensuring a smaller number of parameters, we used DSConv for the down-sampling. The dimensions of the feature map after the last down-sampling are ${\mathrm{X}}^{\mathrm{^{\prime}}}\in {\mathrm{R}}^{{\mathrm{C}}^{\mathrm{^{\prime}}}\times \frac{\mathrm{W}}{16}\times \frac{\mathrm{H}}{16}\times \frac{\mathrm{D}}{16}}$, where ${\mathrm{C}}^{\mathrm{^{\prime}}}=256$.

### Transformer for feature embedding

#### (1) Linear projection and positional embedding

The Transformer cannot directly process the high-dimensional feature map $\mathrm{X}$ that is finally generated by the encoder. This feature map first needs to undergo a linear mapping operation. The linear mapping further extracts features using a convolution layer and reshapes the feature map to 256 patches of dimension $\frac{\mathrm{W}}{16}\times \frac{\mathrm{H}}{16}\times \frac{\mathrm{D}}{16}$. Instead of directly segmenting the original image into patches, the Transformer can model local contextual features in the spatial and depth dimensions of the high-level feature map. The Transformer requires the input to be sequence data, so we use the Reshape operation to flatten the feature map to $256 \times \mathrm{N}$, where $\mathrm{N}=\frac{\mathrm{W}}{16}\times \frac{\mathrm{H}}{16}\times \frac{\mathrm{D}}{16}$.

The importance of the position of each pixel in the image cannot be ignored, and such spatial information is indispensable for the accurate segmentation of tumors. Therefore, we also add learnable position embedding to reconstruct location information.

#### (2) Transformer layer

The architecture of the Transformer layer, which consists of a multi-head attention (MHA) and feed forward network, is shown in Fig. 1.

The MHA consists of eight single attention heads, which can be viewed as mapping a collection of query vectors to output vectors based on key-value pairs. The details are shown in Formulas 1 and 2.

1 $$\begin{array}{c}Multi\,Head\left(\mathrm{Q},\mathrm{K},\mathrm{V}\right)=Concat\left({\mathrm{head}}_{1},\dots ,{\mathrm{head}}_{8}\right){\mathrm{W}}^{\mathrm{O}}\\ where\, hea{\mathrm{d}}_{\mathrm{i}}=Attention\left({\mathrm{QW}}_{\mathrm{i}}^{\mathrm{Q}},{\mathrm{KW}}_{\mathrm{i}}^{\mathrm{K}},{\mathrm{VW}}_{\mathrm{i}}^{\mathrm{V}}\right)\end{array}$$

2 $$\begin{array}{c}Attention\left(\mathrm{Q},\mathrm{K},\mathrm{V}\right)=softmax\left(\frac{{\mathrm{QK}}^{\mathrm{T}}}{\sqrt{{\mathrm{d}}_{\mathrm{k}}}}\right)V\end{array}$$

where ${\mathrm{W}}_{\mathrm{i}}^{\mathrm{O}}\in {\mathrm{R}}^{8{\mathrm{d}}_{\mathrm{v}}\times \mathrm{D}}$, ${\mathrm{W}}_{\mathrm{i}}^{\mathrm{Q}}\in {\mathrm{R}}^{\mathrm{D}\times {\mathrm{d}}_{\mathrm{k}}}$ and ${\mathrm{W}}_{\mathrm{i}}^{\mathrm{V}}\in {\mathrm{R}}^{\mathrm{D}\times {\mathrm{d}}_{\mathrm{v}}}$ are learnable parameter matrices, and Q, K, and V are query, key, and value, respectively.

The Feed Forward Network comprises a fully connected neural network and an activation function.

### Network decoder

Before up-sampling, patches need to be mapped (feature mapping) to the original space, then the up-sampling operation is performed. The decoder also up-samples four times, with the overall operation corresponding to the down-sampling. Because some spatial context information is inevitably lost during down-sampling, we use a skip connection to connect the feature maps corresponding to the down-sampling stage. This skip connection ensures that the new feature maps contain both shallow low-level information and high-level abstract semantic information.

### Comparison details

To verify the effectiveness of our proposed method, we make a comparison with the mainstream medical image segmentation methods. The implementation details of the compared methods are as follows:

- 3D U-Net first constructs a $7\times 7$ convolution block, then constructs four encoder and decoder blocks. Finally, a final convolution block is constructed, including a transposed convolution and two sub-convolution blocks.
- TransBTS is constructed with a series of components. It starts with four encoder blocks, which are then followed by a classical Transformer module containing four Transformer layers, each equipped with eight heads of attention. Subsequently, four decoder blocks are added to the model. To complete the architecture, TransBTS finally incorporates a convolutional layer and utilizes the softmax function.
- ResUNet combines ResNet and U-Net by integrating the residual block in each encoder and decoder block. During skip-connection phase, convolutional blocks are additionally constructed to match the dimensions of the encoder output and the decoder output at the corresponding stage.
- UNet++ aggregates 1 to 4 layers of U-Net together and builds a convolutional layer and sigmoid function at the end.
- The structure of Attention U-Net is generally the same as that of U-Net, with the difference that Attention U-Net adds a layer of attention gates before skip-connection.
- Channel U-Net builds six encoder and decoder blocks and adds the Global Attention Upsample module before skip-connection.

### Training details

All networks were implemented based on the PyTorch framework, and four NVIDIA RTX 3080 with 10 GB memory were used for training. We divided the entire data set into 80% training set and 20% testing set. The testing set is finally used to test the segmentation performance of our proposed model, and the results can be seen in Fig. 1. Given the large size of a single sample, 32 consecutive slices are randomly selected from a sample as input data. We adopted the Adam optimizer in the training process. The weight decay was set to $1\times {10}^{-5}$, the learning rate was $2\times {10}^{-4}$ and $4\times {10}^{-7}$ when the epoch, respectively, reached 0 and 999 (all networks were trained for 1000 epochs), the batch size was set to 2, and the random number seed was set to 1000.

### Evaluation metrics

To evaluate the effectiveness of our proposed method, we used the Dice coefficient (DSC) and Intersection over union (IOU), which are widely used to evaluate the similarity between segmentation results and ground truth data in medical image segmentation. Furthermore, we used the Hausdorff distance and Average surface distance (ASD) to evaluate the similarity of the surface between the segmentation results and ground truth. Mean average error (MAE) is used to assess the absolute error. These metrics are defined in Eqs. 3, 4, 5, 6, and 7, respectively:

3 $$\begin{array}{c}Dice=\frac{2\left|\mathrm{X}\bigcap \mathrm{Y}\right|}{\left|\mathrm{X}\right|+\left|\mathrm{Y}\right|}\end{array}$$

4 $$\begin{array}{c}IOU=\frac{\mathrm{X}\bigcap \mathrm{Y}}{\mathrm{X}\bigcup \mathrm{Y}}\end{array}$$

5 $$\begin{array}{c}H\left(\mathrm{A},\mathrm{B}\right)=max\left(\mathrm{h}\left(\mathrm{A},\mathrm{B}\right),\mathrm{h}\left(\mathrm{B},\mathrm{A}\right)\right)\\ where \,h\left(\mathrm{A},\mathrm{B}\right)=\underset{\mathrm{a}\in \mathrm{A}}{\mathrm{max}}\{\underset{\mathrm{b}\in \mathrm{B}}{\mathrm{min}}\Vert \mathrm{a}-\mathrm{b}\Vert ,h\left(\mathrm{B},\mathrm{A}\right)=\underset{\mathrm{b}\in \mathrm{B}}{\mathrm{max}}\{\underset{\mathrm{a}\in \mathrm{A}}{\mathrm{min}}\Vert \mathrm{b}-\mathrm{a}\Vert \}\end{array}$$

6 $$\begin{array}{c}ASD\left(\mathrm{x},\mathrm{y}\right)={\sum }_{\mathrm{x}\in \mathrm{X}}{\mathrm{min}}_{\mathrm{y}\in \mathrm{Y}}d\left(\mathrm{x},\mathrm{y}\right)/\left|\mathrm{X}\right|\end{array}$$

7 $$\begin{array}{c}MAE=\frac{1}{\mathrm{m}}{\sum }_{\mathrm{i}=1}^{\mathrm{m}}\left|{\mathrm{y}}_{\mathrm{i}}-\mathrm{f}\left({\mathrm{x}}_{\mathrm{i}}\right)\right|\end{array}$$

For statistical analysis, we compare the difference between the prediction results of the proposed method and other methods. We first use the Levene test to check the homogeneity of variance and then conduct Student's t test between our proposed method and other methods.

**Table 3:** Quantitative analysis of different transformer layers

Layers	DSC↑	Hausdorff distance↓	IOU↑	MAE↓	ASD↓
2	0.809	11.473	0.739	0.0006	0.946
**4**	**0.858**	10.996	**0.814**	**0.0005**	**0.509**
6	0.826	**10.412**	0.769	0.0006	1.702
8	0.807	14.721	0.740	0.0007	1.639

## Data Availability

The data used in this study are available from the corresponding author on reasonable request.

## Abbreviations

DSC: Dice coefficient
IOU: Intersection over union
CT: Computed tomography
LRLSM: Localized region-based level set method
ROI: Region of interest
KSVD: K-means singular value factorization
RG: Region growing
KaCNN: Knowledge-assisted convolutional neural network
CNN: Convolutional neural network
DSConv: Deep separable convolution
MHA: Multi-head attention
NLP: Natural language processing
ASD: Average surface distance
MAE: Mean average error
